# Gold Nanozymes: From Concept to Biomedical Applications

**DOI:** 10.1007/s40820-020-00532-z

**Published:** 2020-10-27

**Authors:** Javier Lou-Franco, Bhaskar Das, Christopher Elliott, Cuong Cao

**Affiliations:** 1 Institute for Global Food Security, School of Biological Sciences, Queen’s University of Belfast, 19 Chlorine Gardens, Belfast, BT9 5DL UK; 2 Present Address: Department of Biotechnology and Medical Engineering, National Institute of Technology Rourkela, Rourkela, India

**Keywords:** Gold nanoparticles, Catalysis, Nanozymes, Diagnosis, Nanomedicine

## Abstract

In recent years, gold nanoparticles have demonstrated excellent enzyme-mimicking activities which resemble those of peroxidase, oxidase, catalase, superoxide dismutase or reductase. This, merged with their ease of synthesis, tunability, biocompatibility and low cost, makes them excellent candidates when compared with biological enzymes for applications in biomedicine or biochemical analyses. Herein, over 200 research papers have been systematically reviewed to present the recent progress on the fundamentals of gold nanozymes and their potential applications. The review reveals that the morphology and surface chemistry of the nanoparticles play an important role in their catalytic properties, as well as external parameters such as pH or temperature. Yet, real applications often require specific biorecognition elements to be immobilized onto the nanozymes, leading to unexpected positive or negative effects on their activity. Thus, rational design of efficient nanozymes remains a challenge of paramount importance. Different implementation paths have already been explored, including the application of peroxidase-like nanozymes for the development of clinical diagnostics or the regulation of oxidative stress within cells via their catalase and superoxide dismutase activities. The review also indicates that it is essential to understand how external parameters may boost or inhibit each of these activities, as more than one of them could coexist. Likewise, further toxicity studies are required to ensure the applicability of gold nanozymes in vivo. Current challenges and future prospects of gold nanozymes are discussed in this review, whose significance can be anticipated in a diverse range of fields beyond biomedicine, such as food safety, environmental analyses or the chemical industry.

## Highlights

- The capability of gold nanomaterials to mimic enzyme activities offers new approaches for diagnosis and treatment in the field of biomedicine, which are discussed in this review.
- Controlling the physicochemical properties of the nanomaterials (size, morphology and surface chemistry) remains the first obstacle for endeavouring real-life applications.
- Numerous examples of ex vivo applications in the field of diagnosis are a reality today, whereas further controlling side effects is required for in vivo applications like tumour treatment or intracellular ROS level control.

## Graphical abstract

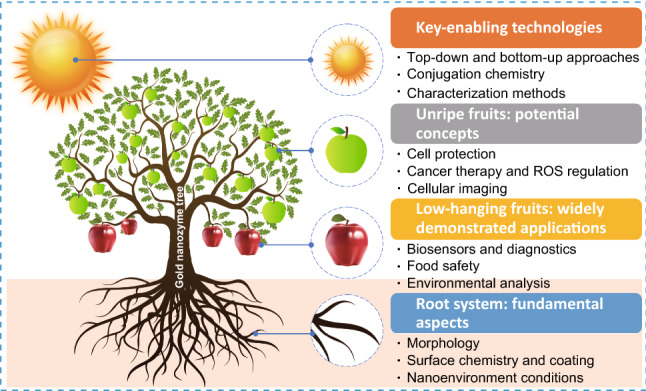

## Introduction

**Fig. 1:**
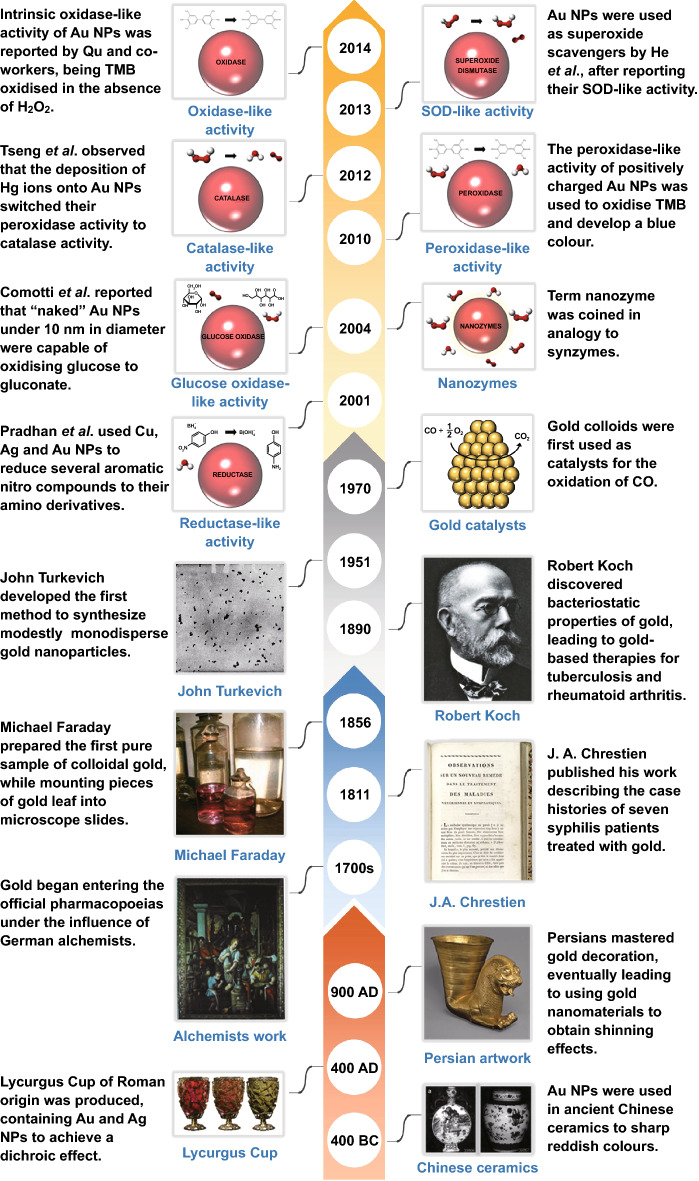
Timeline highlighting relevant historical events that contributed to the development and implementation of gold nanoparticles. Ancient examples are shown before the arrow break, mostly related to the use of AuNPs as an ornament material, whereas biomedical implications are described after the break point. These include John Turkevich’s experiments [9], the first report on the use of gold colloids as catalysts [65] or the first time the term “nanozyme” was used [76]. The yellow arrow shows the years when each nanozyme activity was first reported for gold nanoparticles (reductase [74]-, glucose oxidase [69]-, peroxidase [71]-, catalase [73]-, SOD [72]- and oxidase [68]-like activities)

Colloidal gold nanoparticles (AuNPs) have been widely used for centuries owing to their unique properties not found in their bulk form. Dating back to ancient times (Fig. 1), Persians already knew in the ninth century that metallic nanomaterials could be used to produce special shinning effects on the surface of ceramic materials [1]. Older examples are the Lycurgus cup produced during the Roman time in the fourth century, which contains silver–gold alloy nanoparticles that result in a green–red dichroic effect [2]. Nevertheless, the first information on colloidal gold was reported by Chinese, Arabic and Indian sources from as early as the fifth and fourth centuries B.C. [3]. Since that time, little interest was shown for medical applications until the thirteenth century, when European alchemists started to advocate *potable gold* (i.e., a preparation containing gold particles) for medicinal use [4]. Not many advances were observed for the next 400 years, with some alchemists supporting the medicinal use of gold [5] and some others referring to gold just as “an antidote to poverty” [6]. This impasse started to change in the nineteenth century, when Chrestien [7] published his work that describes the cases of seven syphilis patients treated with gold. In parallel, Michael Faraday produced the first examples of metallic gold colloids, while he was mounting thin sheets of gold leaf onto microscope slides. In 1890, Robert Koch discovered the in vitro bacteriostatic properties of gold, which led to gold-based therapies for tuberculosis and rheumatoid arthritis (thought to be caused by the tubercle bacillus at that time) [8]. All this development culminated with detailed studies of the process of nucleation and growth of colloidal gold carried out by Turkevich et al. [9], which established the basis for the recent boost of gold nanomaterials applied in different fields, including biomedicine.

Despite a wide use of gold colloids throughout history, it was not until 1959 that all the potential implementations of nanomaterials started to be considered, especially after Richard Feynman gave his famous lecture “*There’s Plenty of Room at the Bottom: An Invitation to Enter a New Field of Physics*” [10]. This has led to seminal developments in the field of nanotechnology in the last century, elucidating that properties of materials in the nanoscale differ enormously from the ones found in the bulk material as a result of quantum-sized effects. A large number of excellent studies have reviewed all these specific properties, either physical (optical, electronic, magnetic), chemical (catalytic, supramolecular) or biological (biotoxicity, bioconjugation) [11–14]. These include localized surface plasmon resonance (LSPR), a phenomenon based on the interaction of gold colloids with visible light in which the conduction electrons on the surface of the nanomaterial oscillate in resonance with the frequency of the incident light. Surface plasmons can be used to enhance the sensitivity of several spectroscopic techniques, such as those based on fluorescence and Raman scattering [15, 16]. In addition, novel surface reactivity has been observed in gold nanomaterials, which is at the basis of the catalytic behaviour characteristic of these nanomaterials.

**Fig. 2:**
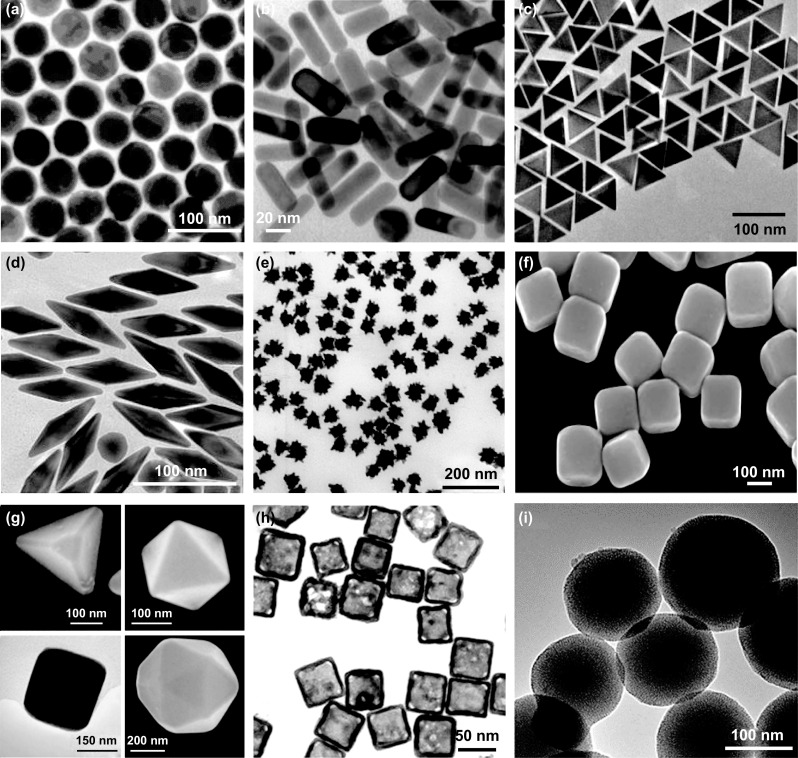
AuNPs synthesized with different shapes following bottom-up approaches: **a** spheres [32]. Copyright 2013 Wiley–VCH Verlag GmbH & Co. KGaA; **b** rods [34]. Copyright 2011 The Royal Society of Chemistry; **c** prisms [37]. Copyright 2015 The Royal Society of Chemistry; **d** bipyramids [42]. Copyright 2016 Fang et al.; **e** stars [38]. Copyright 2008 IOP Publishing Ltd.; **f** cubes [35]. Copyright 2002 Sun et al.; **g** polygon-shaped nanocrystals [40]. Copyright 2004 Wiley–VCH Verlag GmbH & Co. KgaA; **h** cages [39]. Copyright 2010 Wiley–VCH Verlag GmbH & Co. KgaA; **i** gold@silica hybrid nanoparticles [43]. Copyright 2019 Vega et al.

These properties of AuNPs are dependent on their size, shape and surface chemistry. Therefore, controlling these parameters is of paramount importance for the development of AuNP-based chemical and biological sensors. Two approaches are widely used for the synthesis and fabrication of gold nanostructures: bottom-up and top-down. The former relates to techniques such as chemical synthesis, self-assembly or molecular fabrication which involve the nucleation of gold atoms and their growth into colloidal AuNPs from a molecular precursor. The latter refers to methods such as photolithographic micropatternings, pyrolysis and attrition [17]. Micropatterning techniques employ a light, electron or ion beam to selectively pattern nanostructures from precursor resist materials, i.e., photolithography [18], electron beam lithography [19], soft lithography [20], nanocontact printing [21], nanoimprint lithography [22], nanosphere lithography [23], colloidal lithography [24] or scanning probe lithography [25]. Pyrolysis consists in bringing under high pressure and burning a vaporous precursor to generate ashes that are further processed to produce nanoparticles [26–28]. Attrition (or milling) consists in grinding macro- and microscale materials to generate particles of nanosized range [29]. These methods have shown limitations in terms of physical conditions and required equipment, as they are most commonly based on lithography which is neither cost nor time-effective [30, 31]. The high amount of energy required for pyrolysis and the inherent imperfection of attrition methods do not solve the limitations of top-down approaches. Thus, bottom-up approaches have been widely explored to produce AuNPs with a diverse range of sizes and shapes, such as spheres [9, 32], rods [33, 34], cubes [35], prisms [36, 37], stars [38], cages [39], polygons [40] and many others [41, 42] (Fig. 2). This list can be expanded to hybrid gold-related materials [43], self-assembled gold superlattices [44] and many other complexes that have been extensively reviewed [45, 46]. The main advantage of these approaches is that they can produce homogeneous nanostructures with well-defined crystallographic and surface structures. They include chemical reduction, microemulsion, laser ablation, microwave irradiation and green or biological methods [29]. These methods, however, often require reactants that are toxic and can potentially have negative effects in real applications that use gold nanomaterials. In any case, bottom-up approaches have become the preferred method of synthesis in most nanozyme studies. Moreover, unlike other metallic nanoparticles that can also be tuned in different shapes and sizes, AuNPs have shown great stability and rich surface chemistry which allows easy conjugation approaches for biomolecules [47–51]. This versatility together with their attractive optical and electronic properties mentioned above has enabled gold nanozymes to experience a great popularity over other types of nanozymes.

As mentioned, care must be taken when using gold nanomaterials for biomedical applications, as their toxic effects for in vitro and in vivo applications must not be disregarded. Although the bulk gold material is benign and biologically inert, AuNPs in solutions or in aerosols that exhibit mobility (a property that forms toxicity of free nanoparticles) can induce toxicity [52]. This might be caused by small-sized AuNPs which have shown unusual chemical reactivity, especially under 2 nm in diameter, leading to oxidative stress and mitochondrial damage that trigger necrosis [53]. This toxicity, however, very often derives from surfactant or capping agents used during the nanoparticles synthesis, such as cetyl trimethyl ammonium bromide (CTAB) [54–56]. This problem can be addressed by replacing CTAB by biofriendly polymers, such as PEG which has shown little cytotoxicity in vitro and enables a long-lasting circulation in blood due to a stealth character [56]. Larger AuNPs or gold nanostructures being an integral feature of a larger object (the glass or polymer substrate), however, do not show the same problem. Lai et al. [57] prepared microcapsules containing thioridazine, a common antipsychotic, adsorbed on AuNPs to enhance the encapsulation efficiency and obtain a sustained and controlled release of the drug in blood. These oral formulations were administrated to rats with a LD_50_ of nanogold suspension greater than 5000 mg kg^−1^ of body weight, leading to no signs of gross toxicity, adverse pharmacological effects or abnormal behaviour. These results have also been confirmed in vitro by Connor et al. [58], who exposed human K562 leukaemia cells to AuNPs for 3 days and studied the cell viability by MTT assay to determine the survival rate. This study showed that 18-nm AuNPs with various coatings were not toxic in the micromolar range, as opposed to the gold–salt (AuCl_4_) precursor or unwashed CTAB-AuNPs. In vivo studies, on the other hand, require more extensive biodistribution assays to determine where the potential harmful effects may lie. The observed differences are size-dependent in this case, being the smaller ones (15–50 nm in size) accumulated in different tissues like blood, liver, lung, spleen, kidney and stomach, also being able to pass the blood–brain barrier, whereas larger AuNPs were only detected in blood, liver and spleen [59]. Several reviews have tried to shed some light on this matter [60, 61]. It was also suggested that there is no reason to suppose that the immobile nanostructures pose a greater risk for health or environment than larger-scale material, which is gold—a non-toxic material [62]. Nonetheless, any applications of these materials in the biomedical field need to undergo comprehensive safety assessments to determine the physicochemical forms and the concentration at which they would trigger toxicity effects. Safety and toxicity of gold nanomaterials have been substantially reviewed elsewhere [63, 64].

## Catalytic Properties of Gold Nanoparticles

The catalytic properties of AuNPs were already observed and reported in the 1970s [65] and 1980s [66, 67], when gold colloids were used as catalysts for the oxidation of carbon monoxide and proved that they are much more active and stable than conventional catalysts commercially available at that time. Parravano’s group and Haruta et al. first reported these observations, leading the way in this field. Many applications have been explored ever since, as metallic nanoparticles in general and AuNPs in particular possess several catalytic activities [14] (i.e., oxidase [68], glucose oxidase [69, 70], peroxidase [71], catalase [72, 73], superoxide dismutase “SOD” [72] and reductase [74]) (Fig. 1). This has completely changed the perception which would coin bulk gold as “the noblest metal of all” and “the least reactive metal towards atoms or molecules at the interface with a gas or a liquid” [75]. These discoveries led to the term “nanozyme” being coined in the early 2000s by Paolo Scrimin’s group [76], in analogy to the nomenclature of catalytic polymers (“synzymes”) reported by Klotz et al. [77]. This term initially referred to a combination of enzymes and AuNPs, which would offer multiple possibilities for diagnostic assay development [78]. Despite this review focusing specifically in gold nanozymes, it is interesting to note that many of the properties described herein were previously observed in other types of nanomaterials. A clear example of this was set by Yan and co-workers, who first reported that magnetite nanoparticles possess intrinsic peroxidase-like activity comparable to that of an enzyme-catalysed reaction [79]. This pioneer study successfully applied the observed peroxidase-like activity in an immunoassay, paving the way for future gold nanozyme applications.

Since then, the nanozyme activity of different families of nanomaterials has been reviewed. Some of these studies are more generic [80–84], while some others are specific for gold nanomaterials [14], metal–organic frameworks (MOFs) [85, 86] or metal–oxide nanomaterials (MONMs) [87]. Although similar reviews have been published elsewhere, e.g., covering either the factors controlling the activity of gold nanozymes [88] or their potential biosensing and therapeutic applications [89], this review is presented as a comprehensive analysis intending to link both sections, from the fabrication of gold nanozymes, their catalytic mechanisms, potential applications and obstacles in the biomedical field.

Most gold nanozyme studies reported in the literature are based on AuNPs synthesized using bottom-up approaches due to their low-cost and facile syntheses. However, gold nanostructures exhibiting catalytic properties have also been fabricated through top-down approaches [90–92]. Cao et al. [90] found that these properties could be improved by fabrication of nanostructured arrays on Au thin film. Nanosphere lithography followed by oxygen plasma reactive ion etching (NSL–RIE) was the method used to fabricate ordered Au nanoring arrays, giving rise to Au nanopyramids due to the preferential etching of {111} lattice planes of Au. It was observed that their sharp tips along with the Au atoms around the edges provided active sites with high surface energy that could help to overcome the activation energy required for the oxidation of ethanol on the nanoarray surface. Moreover, this study showed that the oxidation of ethanol could follow two pathways, one leading to CO_2_ as a final product and favoured in alkaline medium and the other leading to acetic acid or acetates as final products [90]. Electron beam lithography has also been used to fabricate a nanofluidic device consisting of 11 sets of 5 nanochannels, each decorated with a set of single-Au nanoparticles with identical size. These NPs were grown through Au evaporation and used for the reduction of fluorescein as a model reaction [91]. Also arrays of electrically driven plasmonic nanorods have been used for catalytic purposes, as the numerous resulting tunnel junctions favour the generation of hot electrons, facilitating strongly confined chemical reactions. Oxidation and reduction reactions (such as the oxidation of aromatic amines or the reduction of aromatic nitro compounds) have been studied following this approach, induced by the presence of O_2_ and H_2_, respectively [92]. Despite the diverse possibilities that these approaches offer, the aforementioned limitations for syntheses based on top-down methods have created the perfect environment for bottom-up-based nanomaterials to become predominant in the field of nanozymes.

Scientific efforts have been put together in an attempt to explain this phenomenon, on a proven basis that these chemical reactions take place at the interfacial perimeter around the AuNPs [93]. Thus, several NP parameters such as the size, morphology, surface chemistry or functional layers can strongly affect these catalytic effects, as well as some external parameters such as pH or temperature of the surrounding media. Therefore, understanding the reaction fundaments remains essential to comprehend how all these parameters might affect the efficiency of AuNPs as catalysts.

### Size and Shape Dependence

**Fig. 3:**
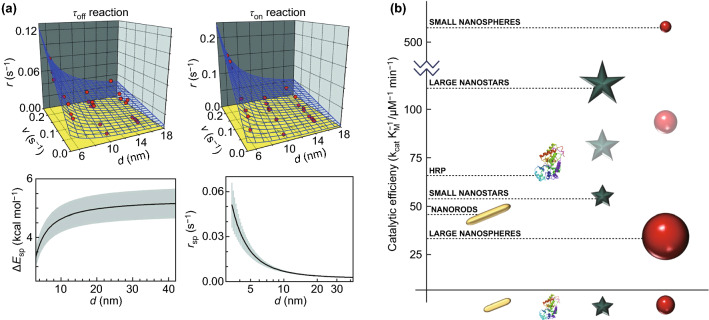
Comparison of the catalytic efficiency of AuNPs with different sizes and shapes. **a** Dynamics of the reduction reaction of resazurin to resorufin catalysed by different-sized AuNPs. It shows how the NP size affects the surface restructuring rate dependence on the rate of turnovers for the catalytic product formation (top left) and for the product dissociation (top right), and the NP size dependence of the activation energy (bottom left) and of the rate of spontaneous dynamic surface restructuring (bottom right) [98]. Copyright American Chemical Society 2010. **b** Comparison of the catalytic efficiency for the peroxidase-like activity of different-shaped and different-sized AuNPs. HRP, their biological counterpart, is also included in the graph

It has been evident that smaller AuNPs usually possess better catalytic properties, which has been attributed to a higher population of low-coordinated gold atoms (corner sites) in the nanomaterial. Such gold atoms were suggested to play a major role in the catalytic activity [94–96]. According to these models, the electron-rich gold plane at the interface would lie behind the observed catalytic properties. As a result, a nanoparticle size of about 2 nm or a height of six atomic monolayers has been found to be optimum for CO oxidation [95]. Therefore, there is a threshold from which a further decrease in NP size will not result in better catalytic activities. This was observed by Lin et al. [97] using AuNPs with sizes ranging from 1.7 to 8.2 nm to reduce p-nitrophenol, resulting in the 3.4-nm-sized NPs in a better catalytic efficiency than the smaller size. Various studies have confirmed this model, using the reduction of resazurin to resorufin catalysed by AuNPs as a model reaction to follow at single-molecule level [98]. Zhou et al. demonstrated that not only the catalytic product formation reaction, but also the product dissociation reaction was affected by the NP size (Fig. 3a). These size-dependent activities are explained from a thermodynamic point of view by changes in the adsorption free energies of the substrate and product, which in turn would be explained by the nanosized effect of AuNPs. According to this explanation, both the unoccupied and the occupied electronic states of the AuNPs increase in energy with a decreasing NP size. Considering that the NP surface acts as electron acceptor as it interacts with the substrate and as electron donor with the product, the mentioned energy increase weakens the NP-resazurin substrate pair and strengthens the NP-resorufin product one. Similar observations were made by He et al., who compared different-sized AuNPs from 2 nm (all atoms are on surface) to 5 nm as catalysts for CO oxidation. The results evidenced the activation of more atoms and the generation of dynamic “single atoms” on surface in smaller Au clusters, which boosts the CO oxidation rate either by a facile transport of CO to the active sites or single-atom catalysis [99].

However, this general size dependence rule cannot be extrapolated to AuNPs with different shapes, despite being true for nanospheres. This is an important remark as very little information has been compiled so far for non-spherical AuNPs. Besides, there is some evidence that the shape of the catalyst affects its properties [100, 101]. Biswas et al. found gold nanorods (AuNRs) of 2.8 aspect ratio to possess a catalytic efficiency for 3,3′,5,5′-tetramethylbenzidine (TMB) oxidation slightly higher than cysteamine-capped gold nanospheres (AuNSs) of 34 nm. However, McVey et al. [102] observed that smaller AuNSs (14 nm diameter) showed a much higher catalytic efficiency, in agreement with the catalytic model studies previously mentioned. Moreover, these smaller AuNSs have overcome the efficiency of biological enzymes (Fig. 3b). Despite lacking further research to explore different shapes and sizes to obtain empirical information, as a general rule, higher surface-to-volume ratios are expected to show enhanced catalytic properties, which would be in accordance with the above explanation related to a higher population of low-coordinated gold atoms in the nanomaterial.

### pH and Temperature Dependence

**Fig. 4:**
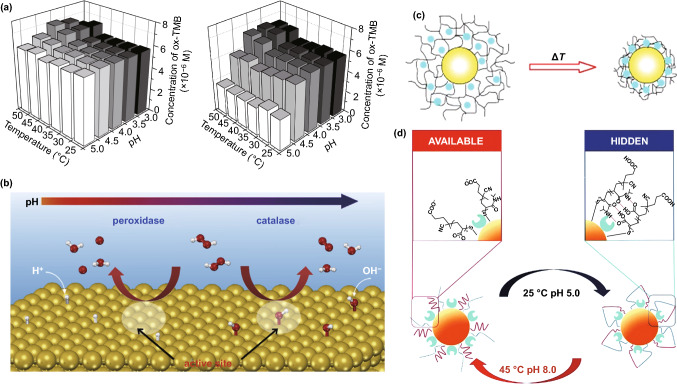
pH and temperature dependence of different catalytic activities of AuNPs. **a** AuNPs coated with different concentrations of the same peptide (3 times higher on the left) will respond differently to temperature and pH changes as for their peroxidase-like capability to oxidize TMB [107]. Copyright 2019 Lee et al. **b** pH switchability of AuNPs in terms of their catalytic activity, which shows different H_2_O_2_ decomposition routes caused by the adsorption of OH groups to the gold surface in basic conditions [108]. Copyright 2015 Elsevier Ltd. **c** Thermosensitive polymeric layers used to stabilize AuNPs might undergo structural changes that block the catalytic active state of the nanomaterials [111]. Copyright 2006 Wiley–VCH Verlag GmbH & Co. KgaA. **d** “On/off” nanozyme controlled by means of a temperature and pH change that result in the formation of a cage-like structure by hydrogen bonding between polymer chains in the surface of the AuNP [112]. Copyright 2019 The Royal Society of Chemistry

Similar to what happens with biological enzymes, pH and temperature also need to be controlled in reactions catalysed by gold nanozymes. For example, TMB oxidation implemented by different AuNPs shows an optimum activity at pH values between 3.5 and 4, whereas its optimum temperature is commonly set between 40 and 50 °C (Fig. 4a) [103–107]. These observations, again, cannot be extrapolated to different peroxidase substrates, i.e., 2,2’-azino-bis(3-ethylbenzothiazoline-6-sulphonic acid) (ABTS) oxidation takes place optimally at lower pH values, while 4-aminoantipyrine (AAP) oxidation is improved at basic pH values [103]. More importantly, some studies have reported that differences in the pH of the media can cause AuNPs to switch their catalytic functionality, potentially leading to the opposite effect. Li et al. observed this by loading AuNPs inside a cell in the presence of H_2_O_2_. In acidic conditions, the peroxidase-like activity was boosted, leading to reactive oxygen species (ROS) generation. When the pH was increased to neutral pH values, the catalase-like activity became predominant and detoxification was the observed effect (Fig. 4b) [108]. The former relates to the base-like decomposition of H_2_O_2_ whereas the latter relates to the acid-like decomposition on the metal surfaces. This pH switchability is triggered by pre-adsorbed OH groups on the gold surface, a feature that only happens at basic pH values as observed by Li et al. [108]. Cao and co-workers have tried to explain the mechanism underlying these observations [71]. They proposed that H_2_O_2_ can be adsorbed on the surface of the AuNPs, being the O–O bond broken up to generate ^**·**^OH radicals. A partial electron exchange interaction between the generated radicals and the AuNPs not only would stabilize the radicals, but also may contribute to the peroxidase-like activity of AuNPs. In the case of the catalase-like activity reported at basic conditions, on the other hand, the OH groups are pre-adsorbed on the gold surface, which become the active sites for the reaction and trigger the acid-like decomposition of H_2_O_2_ into H_2_O and O_2_ [108].

Similar studies have been developed to elucidate the mechanisms of various enzyme-like activities described for AuNPs. Qu and co-workers investigated whether the presence of an active intermediate could explain the oxidase-like activity of AuNPs [68]. It was unambiguously observed that these nanomaterials had the capability to generate singlet oxygen (^1^O_2_), hydroxyl radicals (^**·**^OH) and superoxide (O_2_^−^), which lies behind the oxidase-like properties that AuNPs possess. Likewise, Rossi and co-workers studied the mechanism why complex organic molecules like glucose could be oxidized by AuNPs [109], which is closely related to observations that had previously been done on AuNPs to catalyse the two-electron reduction of dioxygen to convert molecular hydrogen into hydrogen peroxide [110]. Thus, an electron-rich gold species is generated when hydrated glucose interacts with gold surface atoms, which activates molecular oxygen by nucleophilic attack. As a result, a dioxogold intermediate (either Au^+^–O_2_^−^ or Au^2+^–O_2_^2−^) is formed, acting as a bridge for the two-electron transfer from glucose to dioxygen [109]. Regarding their reductase-like activity, it has mainly been described for aromatic nitro compounds being reduced to their corresponding amino derivatives using NaBH_4_. In this case, the substrates are adsorbed on the AuNP surface, while NaBH_4_, a strong nucleophile with high electron injection capability, transfers electrons to the substrate via metal particles [74]. Finally, further investigation is required to explain the catalytic mechanism by which AuNPs scavenge superoxide-mimicking SOD enzymes, which equally involves the adsorption of superoxide onto the AuNPs followed by an electron transfer [72].

The temperature, on the other hand, not only has direct effects on the reaction rate, but also can affect the surface layer that stabilizes the AuNPs. Many polymers used for stabilizing AuNPs might undergo thermodynamic transitions that alter their 3-dimensional structure on the nanoparticle surface (Fig. 4c), leading to a state where reagents cannot diffuse freely to the nanoparticle that acts as catalyst at certain temperatures [111]. This observation has been exploited to create “on/off nanozymes”. Sun et al. immobilized pyrophosphatase on AuNPs, which were subsequently coated with a polymer dually responsive to temperature and pH. The activity of the immobilized enzyme at 45 °C and pH 8 was similar to that of the free enzyme. However, the media conditions switch to 25 °C and pH 5 would favour the formation of interpolymer hydrogen bonding to create a cage-like structure that suppressed the enzymatic activity down to 2% of its initial activity (Fig. 4d) [112].

### Effect of Surface Coating Layers

**Fig. 5:**
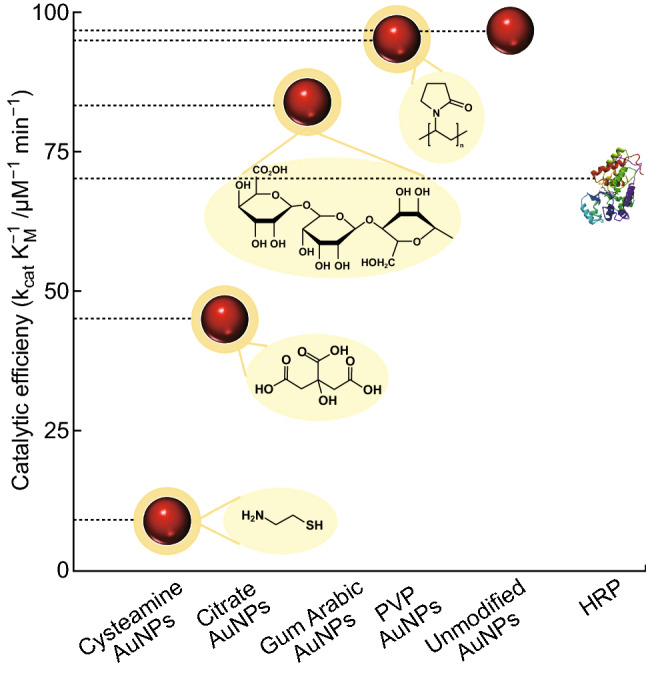
Peroxidase-like activity of AuNP dependence on different surface modifications, measured as its catalytic efficiency [117]

Not only the NP shape and size or external parameters such as pH and temperature, but also any surface coating layers affect the catalytic properties (i.e., surfactants, stabilizing and functional moieties). It has long been understood that thiolates interact with gold surfaces, resulting in the formation of a strong bond [113]. Therefore, most capping agents utilized to stabilize or to coat AuNPs contain a thiol group to bind the AuNPs. Ever since it was first reported, various reviews have collected and analysed the knowledge related to these bonds to understand the formation of self-assembled monolayers of thiolates on metal surfaces [114, 115], where the reason for these catalytic properties suppression may lie. Many studies have proved that the peroxidase-like activity of unmodified AuNPs is significantly reduced when the AuNPs are amino-modified or citrate-capped [116]. These results support the theory of superficial gold atoms as the main contributors to the catalytic effects. Liu et al. have shed some light on the determination of surface accessibility for TMB using AuNPs stabilized with different capping agents (gum Arabic, polyvinylpyrrolidone, citrate, cysteamine and unmodified NPs). The Michaelis–Menten parameters of all these NPs were determined, showing that the catalytic efficiency of unmodified AuNPs outperformed that of horseradish peroxidase (HRP), although the AuNP surface modification could suppress their activity down to 11% of their original activity [117]. These results are illustrated in Fig. 5.

**Fig. 6:**
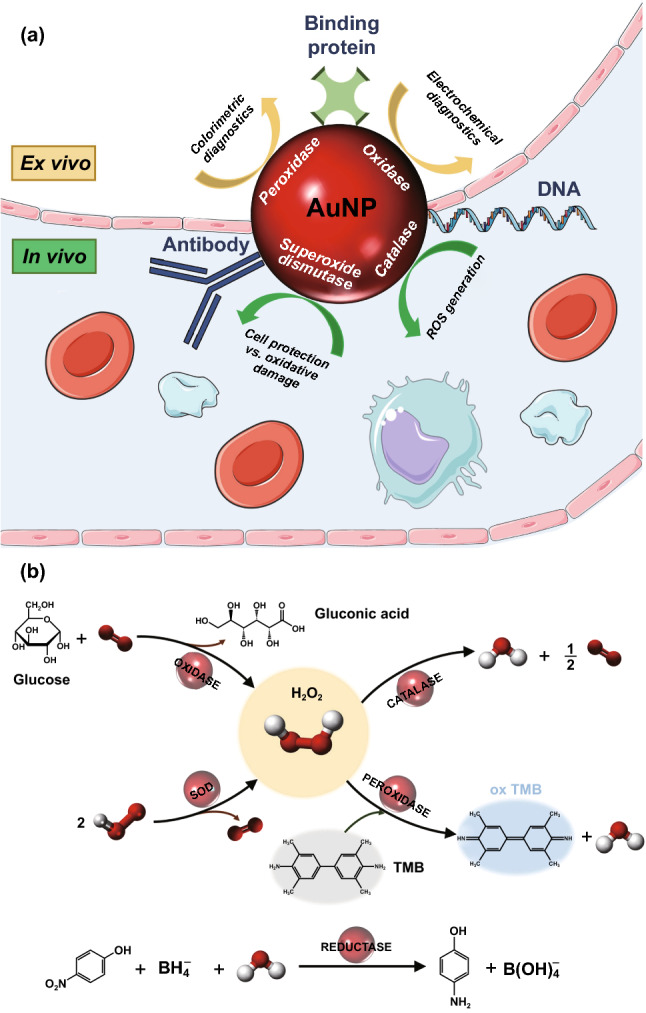
**a** Summary of the different catalytic activities of AuNPs mainly used in biomedical applications. **b** A description of these activities with a representative example

All this knowledge has resulted in numerous biomedical applications, which nowadays start to be exploited from different perspectives, either as diagnostic devices using biological samples or as ROS modifiers for in vivo applications (Fig. 6), which are reviewed in the following sections. Some other applications in the environmental and food sector are also discussed in this review. The methodology followed for the literature review consisted in extracting every research article included in scientific databases such as Scopus and Web of Science, under the search “gold nanozyme” up to January 2020. This resulted in 142 documents that were analysed and classified in an Excel table according to the nature of the nanomaterial used, the catalytic activity exploited, the type of sensor or system developed, the target and the category in which the application could be included (either biomedical, chemical, environmental, veterinary, etc.). Many of these research documents report the fundaments of the nanozyme activity, but the main focus of this review will revolve around developed applications with some direct relevance in biomedicine or indirect implications for health. The potential and limitations of such systems will also be discussed along the text, highlighting the gaps in the field to give readers a complete picture of the state-of-the-art applications of gold nanozymes.

## Biomedical Applications of Gold Nanozymes

Gold nanomaterials have been demonstrated for a wide range of applications in biomedicine, such as photothermal therapy [118], taking advantage of their ability to absorb light and convert it to heat. They have been used as drug delivery vehicles exploiting different loading approaches, such as partitioning [119], surface complexation [120], attachment to capping agents [121], layer-by-layer assembly [122] or even encapsulating drugs inside the AuNPs [123]. Cellular and in vivo imaging using scattering and fluorescence enhancement properties of AuNPs have also been documented [124]. However, the most successful applications in biomedicine relate to the use of AuNPs as visible optical indicators incorporated in lateral flow assays, i.e., the underlying technology used in pregnancy tests. With the discovery of the many nanozyme properties, a new path was unlocked, leading to a wide range of biomedical applications hitherto unexplored. Considering the little information reported so far in vivo for nanoparticles toxicity and distribution, it is not surprising that most applications are related to diagnostics ex vivo, which are reviewed herein.

### Biosensing and Biosensor Developments: Clinical Diagnostics

For many diseases and physiological conditions such as cancer, diabetes or neurodegeneration, the best cure is an early detection that could be achieved by the implementation of biosensors. Many diagnostic tools have already become part of our lives: pregnancy or glucose tests can be bought in pharmacies, whereas antidoping, legal highs or explosive tests are commonly carried out in police controls or at immigration ports. Many other forms of biosensors are being developed and soon will be as common as the previous ones (e.g., detection of clinically important biomarkers such as creatinine and phenylalanine or monitoring of environmental pollutants) [125]. Ever since AuNPs were discovered to possess peroxidase-mimicking activity, they have been used as signal transducers capable to generate a colourimetric signal. Despite the fact that they can catalyse for various substrates such as TMB, ABTS or *o*-phenylenediamine (OPD) [104, 116], gold nanomaterials have mostly been applied for the oxidization of TMB, a substrate catalysed by HRP widely used in immunological assays (i.e., ELISA). Many examples can be found in the literature for sensors applied in biomedicine that use the peroxidase-like activity of gold nanomaterials to oxidize TMB and generate a colourimetric signal [126–132]. Due to the ease of surface modification with specific ligands, gold nanomaterials could allow the development of these diagnostic tools for diverse targets, such as viruses [129–131], exosomes [133], red blood cells [127], amino acids or even ions [126]. Apart from coloured products, gold nanozymes can catalyse for the conversion of different substrates into fluorescent, luminescent or Raman active products. Alternatively, some systems use the electrons produced in the reaction to develop electrochemical sensors. All these possibilities are discussed in this section.

**Fig. 7:**
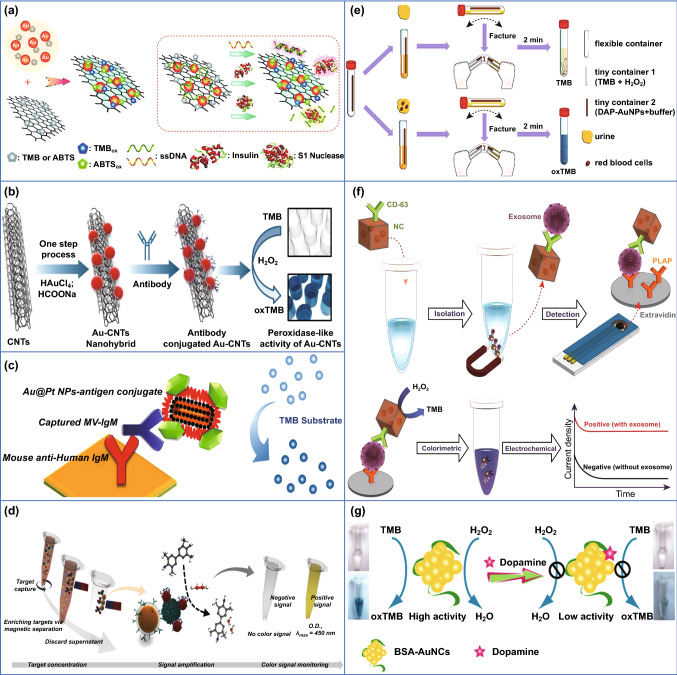
Peroxidase-like activity of AuNPs used as clinical diagnostic tools. **a** AuNPs grown on graphene sheets used to develop a “off–on” colourimetric sensor [134]. Copyright 2012 American Chemical Society. **b** Nanohybrid composed of AuNPs and CNTs acting as colourimetric influenza virus detection [131]. Copyright 2016 Elsevier B.V. **c** Rod-shaped Au–Pt core/shell NPs used for measles virus detection [129]. Copyright 2018 Long et al. **d** Magnetic nanobeads combined with AuNPs for separation and detection of influenza virus [130]. Copyright 2018 American Chemical Society. **e** User-friendly device for haemoglobin and red blood cells detection in urine samples [127]. Copyright 2018 Elsevier B.V. **f** Gold-loaded nanoporous ferric oxide NPs used for direct exosome detection [133]. Copyright 2019 American Chemical Society. **g** Fluorometric and colourimetric detection of dopamine based on AuNCs [141]. Copyright 2012 Elsevier B.V.

Hybrid systems based on Au with other nanomaterials could lead to an enhanced catalysis. The applicability of this phenomenon in the biosensor field was first reported by Liu et al., who described the synergism between catalytic active graphene and AuNPs to detect specific DNA molecules (Fig. 7a) [134]. A similar strategy was followed by Ahmed and co-workers, who immobilized AuNPs on the surface of carbon nanotubes to enhance the peroxidase-like activity of the resulting nanohybrid, which developed a more intense colour in less time. This system (Fig. 7b) could effectively detect influenza virus A (H3N2) in human serum with a sensitivity of 3.4 PFU mL^−1^ (i.e., plaque-forming units per mL) [131], 100 times higher than reported conventional ELISA assays and 500 times higher than commercial immunochromatography kits (ImunoAce Flu, TAUNS Laboratories, Inc., Numazu, Shizuoka, Japan). These synergistic effects for oxidizing TMB were also observed with Au–Pt core/shell nanorods for detection of measles virus (Fig. 7c) [129]. Furthermore, Oh et al. [130] developed a different sensor including a capturing step with magnetic nanoparticles for the detection of influenza virus A (H1N1) in human serum (Fig. 7d). Following this approach, a similar limit of detection (LOD) was observed without making use of synergistic catalytic effects. Several studies have attempted to explain the mechanism by which the synergistic effect occurs. Liu et al. [134] proposed that the attractive interaction between carbon 2p (from reduced graphene) and Au 5d (from AuNP) orbitals around the carbon vacancy and defect would explain not only the strong covalent interfacing, but also the observed favourable H_2_O_2_ adsorption, thus enhancing its reduction. Similar charge transfer mechanisms have been proposed to explain this synergism in bimetallic nanoparticles [135].

Due to their simplicity and unnecessity of laboratory-based requirements, all these systems have a clear potential to become portable sensors and to be applied for point-of-care (POC) diagnostics. A good example has been developed by Wu et al. (Fig. 7e) to detect occult blood in urine, commonly linked to a serious health condition such as chronic nephrotic syndrome or urinary system tumours. It consists of a flexible container with two tiny containers inside it, one with TMB and H_2_O_2_ reagents for the colourimetric reaction and the other with buffered AuNPs. After adding the urine sample to the flexible tube this is bended to break the tiny containers inside and develop the colourimetric reaction only in the presence of Fe^2+^ ions, which restore the peroxidase-like activity of AuNP capped with a purine derivative, otherwise remaining inactive. This system permits to detect either haemoglobin or red blood cells presence in urine due to their high content in Fe^2+^, by using a colour chart that enables a fast and visual detection [127]. This system, however, remains qualitative, providing a semi-quantitative estimation in the best-case scenario. This limitation could be circumvented using a smartphone camera, which would permit to quantify the colour intensity by means of a calibration curve previously built. It is curious to see that, to date, only one publication [136] is obtained when the terms “gold nanozyme” AND “smartphone” are searched in Scopus database. The proposed method to detect mercury ions in environmental water samples is further discussed in Sect. 3.1.

Gold nanozymes are used for the development of not only colourimetric sensors but also electrochemical detection platforms. The electrochemical approaches have been shown to have great repeatability, accuracy and robustness, while advancements in miniaturization have made the design of portable and quantitative sensors possible. Boriachek et al. [133] used gold-loaded ferric oxide nanocubes functionalized with an exosome-associated antibody to allow magnetic isolation of the captured target, whereas the peroxidase-like activity of the encapsulated AuNPs could generate a colourimetric signal. Abreast of the colourimetric output, the reaction happened on a screen-printed carbon electrode; therefore, the electroactive diimine generated during the TMB oxidation process [137] could also be quantified through chronoamperometry (Fig. 7f). In the same line, other research groups have used screen-printed gold electrodes modified with aptamers specific for cardiac troponin I, a well-known standard biomarker for the early detection of Acute Myocardial Infarction [138]. This system uses bimetallic Cu@Au nanozymes (capable of oxidizing hydroquinone to benzoquinone) immobilized on magnetic metal organic framework nanocatalysts. Similar to other synergistic effects described above, the outstanding catalytic activity of the resulting nanohybrid allows highly sensitive detection through differential pulse voltammetry.

Finally, gold nanozymes can also be harnessed in other sensing approaches based on fluorimetry and spectroscopy for the detection of urinary spermine [139], HIV and HCV [140] or dopamine [141] (Fig. 7g). Due to complicated optical components required, the spectroscopic methods often lack portability; thus, their development and practical implementations are more limited. In case of surface-enhanced Raman spectroscopy (SERS)-based systems, Hu et al. developed a system that uses the peroxidase activity of AuNPs to detect the lactate and glucose levels in living rats’ brains, which are associated with ischaemic stroke. While there are some portable Raman spectrometers available on the market (such as QE Pro-Raman by Ocean Insight or TruScan™ GP analyser by ThermoFisher), there is still a long way for engineering and optimizing the systems for POC diagnostics.

Together with a vast range of approaches for surface modifications and bioconjugations, it is evident that enzyme-mimicking activities of AuNPs and Au nanohybrid structures can allow versatile biosensor platforms generating either a colourimetric, electrochemical, fluorescent or spectroscopic measurable signal. As it is a new field, only a few examples have been described in the literature regarding gold nanozymes applied for clinical biosensors used in real samples. A summary is presented in Table 1.

**Table 1:** Clinical biosensors relate the use of the catalytic properties of gold nanomaterials

Target	Nanomaterial	Activity	Outputs	Sample	LOD	References
Haemoglobin & red blood cells	AuNPs	Peroxidase	Colourimetric	Human urine	0.96 nM and 1.6 × 10^6^ cells L^−1^	[127]
Heparine and heparinase	Gold nanoclusters	Peroxidase	Colourimetric	Diluted serum	0.3 and 0.06 µg mL^−1^	[106]
Cu^2+^ and histidine	Gold nanoclusters	Peroxidase	Colourimetric	Human serum, blood	0.1 nM and 20 nM	[126]
*Pseudomonas aeruginosa*	AuNPs	Peroxidase	Electrochemical	Drinking water	60 CFU mL^−1^	[187]
Placental cell-derived exosomes	Au-loaded Fe_2_O_3_ nanocubes	Peroxidase	Colourimetric, electrochemical	Cell culture media	8 × 10^6^ vesicles per million cells per 24 h (10^3^ exosomes mL^−1^)	[133]
Spermine	Ag–Au bimetallic NPs	Oxidase and Peroxidase	Fluorescence	Urine	0.87 nM	[139]
Rubella virus (IgM antibodies)	Au–Pt core/shell nanorods	Peroxidase	Spectroscopic, colourimetric	Human serum	10 ng mL^−1^	[225]
Cysteine and homocysteine	Au–Pt NPs	Peroxidase	Spectroscopic, colourimetric	Human serum	3.5 and 1.6 nM	[128]
Measles virus (IgM antibodies)	Au–Pt core/shell nanorods	Peroxidase	Spectroscopic	Human serum	10 ng mL^−1^	[129]
Influenza virus A (H1N1, H3N2)	AuNPs and magnetic nanobeads	Peroxidase	Spectroscopic, colourimetric	Spiked samples and human serum	4.42 × 10^−14^ g mL^−1^ and 2.5 PFU mL^−1^	[130]
Cocaine	Multi-shaped AuNPs and ZnSeS alloyed quantum dots	Peroxidase	Spectroscopic	KCl–HCl buffer	112 nM	[226]
HIV and HCV DNA	Pt–Au bimetallic NPs	Oxidase	Fluorescence	Buffer	5 pM	[140]
Glucose and lactate	AuNPs@MOF	Peroxidase and Oxidase	SERS	Living rats’ brain	4.2 and 5 µM	[227]
Murine norovirus	AuNPs	Peroxidase	Colourimetric	Human serum, shellfish homogenate	200 PFU mL^−1^	[186]
Dopamine	Au nanoclusters	Peroxidase	Fluorescence, colourimetric	PC12 cells	10 nM	[141]
Xanthine	Au nanoclusters	Peroxidase	Spectroscopic	Urine and serum	20 nM	[228]
Influenza virus A (H3N2)	AuNP–CNT hybrid	Peroxidase	Spectroscopic, colourimetric	Human serum	3.4 PFU mL^−1^ (10 PFU mL^−1^ in real samples)	[131]

### ROS Generation

Gold nanozymes are not only applied for sensing devices, but can also be used as drug agents for therapeutics. This application has been mainly linked but not limited to small AuNPs (less than 2 nm in size), which are capable of penetrating the cells and cellular compartments and cause cytotoxicity (necrosis) by means of oxidative stress [142]. Thus, nanozymes with peroxidase-mimicking activity can catalyse the hydrogen peroxide decomposition and produce ROS [143–145]. The induced toxicity has been mainly used for tumour treatment, by first directing the nanoparticles to the targeted area and then enhancing their catalytic properties through photodynamic therapy (PDT). Despite not being a perfect photoregulation system, with clear on/off states, this approach can help reduce the secondary effects in non-targeted body tissues. Therefore, different nanocomplexes can be introduced in the body through the preferred administration via and after they are accumulated in the area of interest, the ROS generation process can be improved. This approach requires a smaller nanomaterial concentration to produce the same effect than the non-enhanced system, thus decreasing the drug concentration that needs to be administrated and the side effects caused by ROS generation elsewhere. However, very important external parameters govern nanozyme activities. Not only controllable parameters such as size, shape or nanoparticle surface modification, but also some others determined by the biological matrix, such as pH, temperature or the presence of interfering molecules [146]. Therefore, it becomes very important to understand the tumour microenvironment if gold nanozyme properties are to be used for a clinical treatment. Well-known characteristics such as acidity [147], hypoxia [148] and abnormally high concentrations of H_2_O_2_ [149, 150] in the affected area are a common denominator of the disease.

**Fig. 8:**
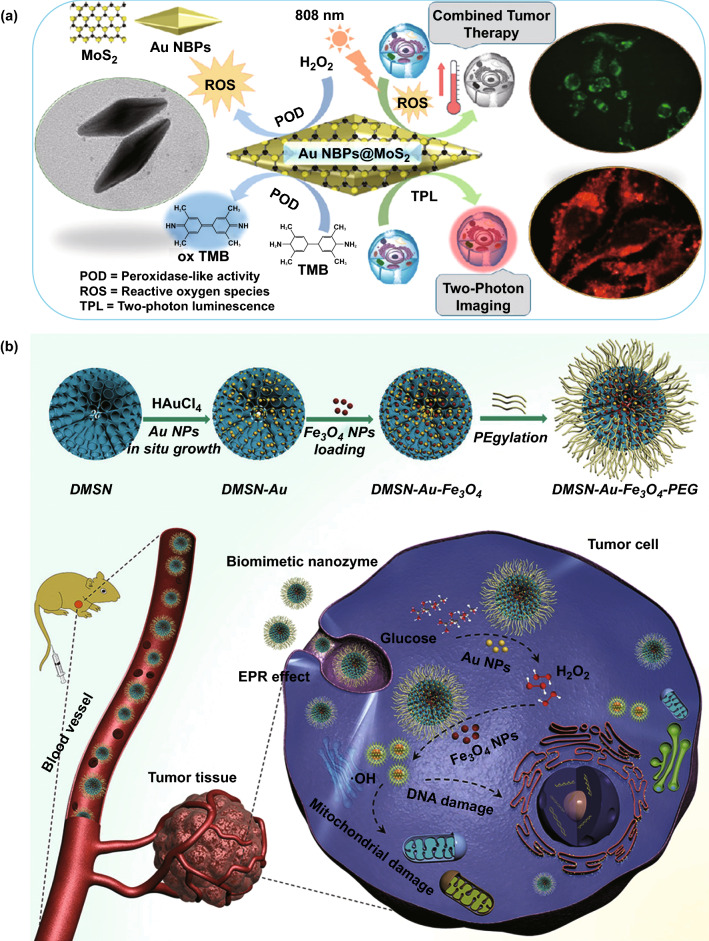
Gold nanozymes applied for tumour therapy. **a** Peroxidase-like activity is combined with photodynamic and photothermal therapies for tumour imaging and localized treatment [155]. Copyright 2018 American Chemical Society. **b** Various enzymatic activities are combined to generate ROS inside the cell after directing the NPs to the affected tissue [157]. Copyright 2018 Wiley–VCH Verlag GmbH & Co. KGaA

Conventional PDT involves light and a photosensitizing chemical substance, which combined with molecular oxygen, leads to phototoxicity. A recent approach used platinum nanoparticles (PtNPs) decorated on photosensitizer integrated metal–organic frameworks for enhanced PDT [151]. The nanoplatform successfully used the catalase-like activity of PtNPs to convert the abundant H_2_O_2_ in the tumour region into O_2_, thus modifying the hypoxic environment and facilitating the light-irradiated formation of cytotoxic ^1^O_2_. This was the first study that exploited the nanozyme activity of metallic NPs to achieve an enhanced PDT. Different gold nanozymes have been combined with PDT ever since, paving the way for a new promising approach entirely to explore. Moreover, any laser wavelength within the therapeutic window (i.e., 650-1350 nm) can be used for this enhancement purpose, being one or another more convenient depending on the absorption profile of the nanocomplex being used. This way, 808-nm light irradiation has been used to improve the ROS generation of AuNPs embedded in porous hollow carbon shell nanospheres [143, 152], whereas other studies have used 660-nm irradiation to increase the method efficiency with AuNPs embedded in a zeolitic imidazolate framework [153] or 671 nm wavelength for hollow mesoporous organosilica nanoparticles containing AuNPs [154]. Interestingly, different nanomaterial combinations have been used, leading to different paths for generating ROS in which AuNPs can take diverse roles. Fan et al. pioneered the application of AuNPs for thermal cancer therapy combined with nanozyme activity. They encapsulated AuNPs in hollow carbon shell nanospheres and showed that, under an acidic environment, they retained the ability of ROS generation. More importantly, this process could be photoenhanced by the 808-nm laser irradiation, substantially inhibiting CT26 tumour growth in vivo [143]. This capability of ROS generation by AuNPs was confirmed by Zhang et al., who delved into the matter by using mesoporous carbon nanospheres doped with AuNPs as a drug delivery system. This nanocomplex was loaded with dye IR780 to enhance the photothermal therapy, so that when the tumour tissue was irradiated with the 808-nm laser it would also generate heat, thus further enhancing the photothermal therapy. Together with the nanozyme properties of AuNPs to decompose H_2_O_2_ located in the tumour cells to ^**·**^OH and cause intracellular oxidative damage, this synergistic strategy has demonstrated favourable results as a therapy for folate-overexpressed gastric cancer tumours [152]. Many others [155, 156] came simultaneously to a conclusive consensus: the combination of photodynamic and photothermal therapies provides a novel approach with excellent synergistic therapeutic outcomes (Fig. 8a). However, some other tumour treatments take advantage of different gold nanozyme properties (Fig. 8b), either using their glucose oxidase activity [154, 157] or their catalase activity [153] to alleviate the pernicious effects. The former system used a nanocarrier loaded with collagenase to degrade the collagen I fibre in the extracellular matrix and enhance the penetration of the system and O_2_ infiltration, AuNPs capable of oxidizing glucose and generate H_2_O_2_ as a by-product and copper-based complexes to generate ROS. The latter addressed tumour hypoxia through the catalase-like activity observed in AuNPs, which takes advantage of the abundance of H_2_O_2_ in tumour areas to generate O_2_ that can be used by a photosensitizer to increase ROS levels. It is interesting to note that while different nanozyme activities derived from the same AuNPs can be boosted or inhibited, it is just likely that they coexist up to some points. Again, the microenvironment and the surface properties of the nanocomplex will play a crucial role in this sense. No studies have been found, however, clarifying whether these synergistic effects have any impact in the final outcomes for tumour treatment.

Likewise, ROS generation has also been used for bacterial inactivation and wound disinfection [68, 158]. Qu and co-workers first reported that AuNPs embedded in mesoporous silica (MSN-AuNPs) could act as oxidase mimics [68] and were capable of generating ROS that led to antibacterial activity. This approach showed that MSN-AuNPs served as efficient and safe antibacterial agents even in the absence of H_2_O_2_, a powerful oxidizer which in high concentrations harms healthy tissues and delays wound healing. Microscopic analyses of *Staphylococcus aureus* and *Escherichia coli* showed that ROS generated by MSN-AuNPs could oxidize and destroy the bacterial lipid membranes, thus inhibiting the growth of both Gram-positive and Gram-negative bacteria. Further applications of MSN-AuNPs using *Bacillus subtilis* biofilms were investigated, whose antibacterial activity also resulted in an effective breakdown of existing biofilms and prevention of new biofilm formation. This was followed by a similar study, using AuNPs with ultrathin graphitic carbon nitride to take advantage of their synergistic effect and catalyse the decomposition of H_2_O_2_ to ^**·**^OH radicals in an efficient manner [158]. Importantly, the mentioned synergism enables to use lower concentrations of H_2_O_2_ to reach the required antibacterial activity, thus avoiding the side effects mentioned above. This system has been applied for bacteria killing, biofilm degradation, wound disinfection and lung infection treatment. In vitro experiments showed the nanocomplex to have bactericidal activity against both Gram-negative and Gram-positive bacteria, as well as high efficiency in breaking down biofilms and preventing new ones to be formed. These approaches provide a novel alternative to tackle the growing healthy issue caused by persistent infections [159].

In addition to the applications described, AuNPs have also been used in conjunction with α-FeOOH/porous carbon as biomimetic catalyst for Fenton reaction [160]. The proposed system used the glucose oxidase activity of AuNPs to generate gluconic acid, which helps to adjust the microenvironmental pH to promote the Fenton reaction and H_2_O_2_ to induce its reaction with Fe^2+^, thus generating ^**·**^OH radicals. This reaction has many applications, especially for organic wastewater treatment [161], as the ROS formed can quickly and non-selectively degrade most organic pollutants to CO_2_ and H_2_O. Correspondingly, any novelty that improves its yield could have a direct application in different sectors of the environmental field.

### Cell Protection

Interestingly, AuNPs could also be used for ROS protective effects [162–165]. It might seem contradictory to what has just been explained, but as mentioned before, the nanozyme activity of AuNPs can be tuned or modulated depending on the surface chemistry of the nanoparticle or the microenvironment in which they are being used. This duality has been exploited to develop antioxidant nanocomplexes for protecting certain tissues from harmful effects caused by a high ROS presence. This is important since ROS, which are known to be involved in the regulation of gene expression and cell signalling cascades, can cause serious damage within biological systems of the human body if present at high concentrations [166]. For instance, a build-up of ROS within the body can induce an inflammatory-type response leading to diseases such as chronic obstructive pulmonary disease (COPD) [167]. Additionally, brain tissue damage and central nervous system-related disorders are associated with ROS, being the attenuation of oxidative stress a promising strategy to prevent conditions such as Parkinson, Alzheimer or amyotrophic lateral sclerosis [168, 169]. Most approaches propose to use antioxidant enzymes to revert this situation, but their delivery to the brain has shown major challenges, including proteolytic degradation, immunogenicity, short circulation half-time and poor permeability across the blood–brain barrier [168]. All these drawbacks open the way for alternative strategies using enzyme mimetic AuNPs as ROS scavengers.

Kunjiappan et al. [162] reported on these applications by using biologically synthesized AuNPs from *Azolla microphylla* extracts as antioxidant and hepatoprotective tools. This in vitro study compared the cell viability (among other parameters) of primary hepatocytes treated with acetaminophen (APAP) in the presence and absence of AuNPs. The study concluded that AuNPs presented antioxidant and hepatoprotective effects in APAP-treated hepatocytes by inhibiting ROS generation, scavenging free radicals and increasing the antioxidant defence enzymes. However, this empirical study did not prove the mechanisms underlying the effects observed and lacked some in vivo experiments to prove its applicability.

**Fig. 9:**
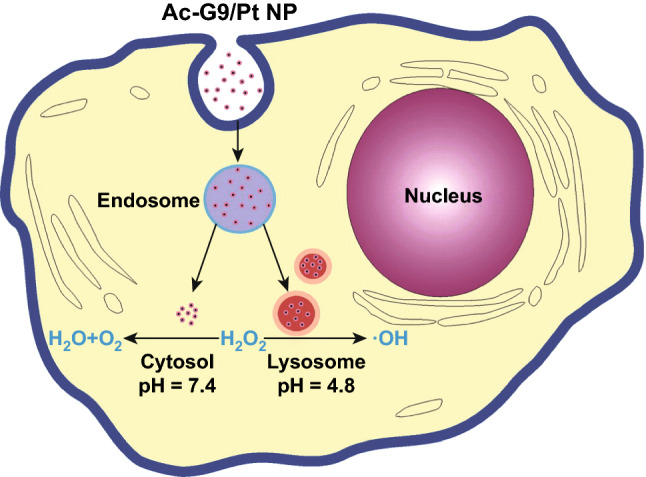
pH dependence of the catalytic properties of gold nanozymes shows two decomposition routes for H_2_O_2_. Acidic media promote the splitting of H_2_O_2_ into two hydroxyl radicals (peroxidase-like activity), whereas neutral pH media decompose it into H_2_O and O_2_ (catalase-like activity) [171]. Copyright 2013 American Chemical Society

**Fig. 10:**
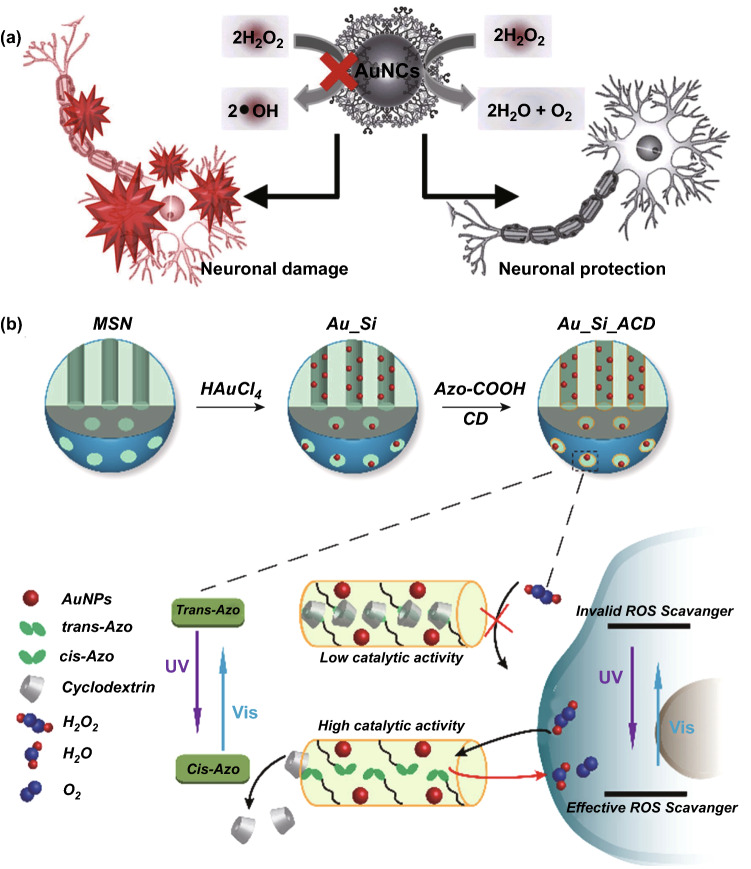
Gold nanozymes used as ROS scavengers for cell protection. **a** Catalase-like activity of amine-terminated dendrimer-entrapped AuNCs used for neuronal protection against oxidative damage [164]. Copyright 2016 Wiley–VCH Verlag GmbH & Co. KgaA. **b** Light-mediated reversible modulation of ROS levels using mesoporous NPs loaded with AuNPs [163]. Copyright 2017 Wiley–VCH Verlag GmbH & Co. KGaA

On the basis of previous works using iron oxide NPs [170] and platinum NPs [171], it had been observed that several nanozymes exhibited pH-switchable catalase-like and peroxidase-like activities (Fig. 9). This effect was later demonstrated in other metallic NPs, such as gold and silver [108], being the adsorption energy between the metals and H_2_O_2_ the key aspect to favour one or the other reaction. Since the adsorption process is affected by the pH of the media, this explains the pH-switchable activity empirically observed. It is based on this knowledge that Liu et al. published their work to shed some light on how to circumvent the pH dependence by simply modifying the NP surface. They discovered that gold nanoclusters (AuNCs) deprived of their peroxidase-like activity while retaining their catalase-like activity under different pH values if modified with amine-terminated poly(amidoamine) (PAMAM). It was suggested that 3°-amines on the surface of the NP provided sufficient suppression of the critical mediator ^**·**^OH [164]. This system was used as a primary neuronal protection against oxidative damage (Fig. 10a), after demonstrating that mice neurons did internalize the AuNCs by endocytosis and that these neurons showed a significantly higher survival rate when treated with H_2_O_2_. Other approaches have used a light-mediated modulation system to control ROS levels in living cells, giving rise to smart nanozymes (Fig. 10b). This was achieved by encapsulating AuNPs in azobenzene (Azo) decorated mesoporous silica NP. Under visible light, cyclodextrin (CD) interacts with Azo through host–guest chemistry, blocking the catalytic sites of the nanocomplex and inhibiting its catalase-like activity at neutral pH conditions. However, UV illumination causes an isomerization of Azo to its *cis* conformation, resulting in CD being released from the system and recovering the catalase-like activity to become an effective ROS scavenger [163]. This turn on/off system was tested in MCF-7 cells, whose viability was modified at will depending on the light exposure. It is noteworthy that this system has some limitations, such as a relatively low nanozyme activity in the experimental conditions or low tissue penetration capacity of UV light. This approach resembles the PDT treatments before mentioned, arguably going a step further in terms of distinction between the two possible states. Be this as it may, there is no doubt that all these systems have paved the way for a whole new generation of smart nanozymes. Countless benefits may derive from these protective effects, not only for health purposes, but also for any process involving cell viability, such as in vitro fertilization (IVF). Dashtestani et al. [165] developed apoferritin-containing gold–silver NP to be used as ROS scavengers. The nanozyme showed both SOD- and catalase-like activity, thus decomposing superoxide ions to hydrogen peroxide, which subsequently is converted into water and molecular oxygen. This nanozyme was examined as a protective agent during the cryopreservation of human sperm, to control the freeze–thaw induced oxidative stress. The results showed a significant increase in the sperm motility and viability, being both parameters essential for sperm–egg fusion and fertilization.

## Other Applications of Gold Nanozymes

Different options have been discussed throughout the review related to treating or diagnosing a certain biomedical condition. Conversely, little importance is often given to other matters that threaten our health in an indirect manner, such as environmental or food contamination. As a result, these conditions tend to fall by the wayside and only sporadic food- or water-related outbreaks remind us the potential risks of not diagnosing a disease in our environment. Fortunately, there is a high awareness of this within the scientific community, as our group previously reported in a comprehensive and interactive platform that reported over 900 sensors retrieved from the scientific literature and commercial market for aquatic toxins, mycotoxins, pesticides and micro-organism detection [172]. Furthermore, the catalytic properties of AuNPs can be applied to different bioprocesses with industrial or scientific interest, either for oxidizing amino acids [173], cleaving RNA [174], inducing the Fenton reaction [160] or even to catalyse enantioselective reactions [175]. Despite the fact that all these reactions can be carried out by biological counterparts, AuNPs may provide some key advantages during the process that sometimes can lead to lowering the production costs. These and other biological aspects of gold nanozymes, not strictly related to biomedicine, are discussed in this section.

### Environmental Applications

Most applications encompassed in this category are related to diagnostic tools to detect toxic contaminants in environmental waters. This mainly includes heavy metals [136, 176–183], pesticides [100, 184, 185] or micro-organisms [102, 186–189], for being the most common contaminants, but it could potentially include any others. Nonetheless, these are not the only examples of gold nanozyme applications in the environmental sector, especially since their catalytic activities started to be exploited for wastewater treatment to degrade or convert toxic molecules into innocuous products [190, 191]. It is important to note that many of these contaminants are anthropogenic. One obvious example is the E-waste, which comprises discarded electronic devices. It was estimated that 44.7 million tonnes of E-waste were generated globally in 2016, leading to lots of contaminants being released to the environment such as heavy metals (Pb, Sb, Hg, Cd, Ni), polybrominated diphenyl ethers (PBDEs) and polychlorinated biphenyls (PCBs) [192]. Moreover, the transfer of E-waste to ground and surface water, agricultural soils, rice, fish and humans has been proved [193]. A constant exposure to these contaminants brings harmful health effects with it. Far from being reduced, E-waste numbers are expected to keep growing, thus increasing the health threat.

**Fig. 11:**
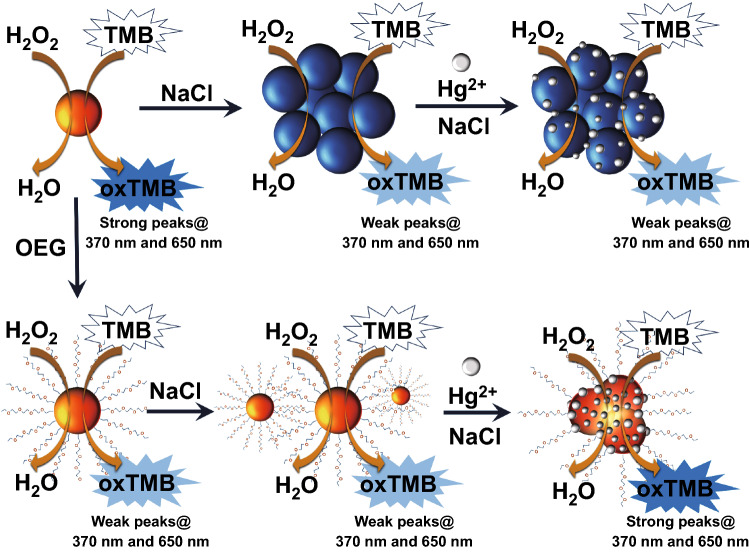
Colourimetric sensor using gold nanozymes for the detection of Hg^2+^ ions in seawater samples [183]. Copyright 2020 Logan et al.

Moving to the detection mechanisms themselves, the peroxidase-like activity of gold nanozymes is again the preferred approach for developing a sensor. Interestingly, many sensors for Hg detection have been reported in the literature using AuNP nanozyme activity [136, 176–178, 180]. The reason behind it is that mercury can form an Au–Hg amalgam on the surface of the AuNP that enhances its peroxidase-like activity. This system has proved to be very sensitive and, more importantly, selective towards Hg. Slight differences can be found between all these systems, whose sensitivity ranges between 0.15 and 6 nM. The best LOD was found using an Au/Fe_3_O_4_/GO hybrid material that not only could detect Hg^2+^ in river and tap water samples, but would also allow for an efficient and quick Hg removal by deposition of Hg^0^ on the nanohybrid surface and its subsequent collection using an external magnetic field [177]. On the other hand, higher LOD (40 times less sensitive than the previous example) corresponds to a portable and paper-based approach that can be measured using a smartphone camera [136]. This approach has the particularity of being the only one that combines gold nanozymes with smartphones in any way. Recently, Logan and co-workers have lowered the LOD values reported for Hg detection using highly stable oligo ethylene glycol (OEG)-functionalized AuNPs [183], whose nanozyme activity was recovered in the presence of the metal (Fig. 11). A tenfold increased sensitivity was observed using this approach, which reached 10 pM in tap and bottled water, while the sensitivity for saline solution was 65 pM. Despite most sensors described in this section target Hg ions, other systems have been developed to detect specifically Ce^3+^ [194] making use of its redox recycling ability to enhance TMB oxidation or Ag^+^ [195] taking advantage of its ability to inhibit the nanozyme activity after being reduced onto AuNPs.

Other nanozyme sensors have been developed for malathion detection, a widely used pesticide in agriculture. These make use of the sulphanyl group from malathion molecules and its strong affinity for AuNP surfaces, thus quenching their peroxidase-like activity [185]. This inhibitory approach was first tested with AuNR [100], reaching a LOD of 1.78 µg mL^−1^ (though 10 times higher than the permissible levels of malathion in water). Then, a similar system using palladium–gold bimetallic nanorods [185] managed to lower the LOD down to 60 ng mL^−1^, well below the permissible levels, showing no cross-reactivity with analogous organophosphates or metal salts despite the lack of a biorecognition element in the system. Again, the synergistic effect of nanohybrids shows the great potential they have in this field.

Alternatively, gold nanozymes can be used for water treatment instead of diagnostics. This sometimes involves catalytic properties from AuNPs that have barely been mentioned herein: reductase properties. Such a system becomes a quite interesting approach for reducing the toxicity of water contaminants in industrial wastewaters before they are released to the environment. p-Nitrophenol (PNP) is a contaminant susceptible to this approach. It is widely used in petrochemical synthesis and its presence in industrial wastewaters might lead to liver and kidney damage, anaemia or systemic poisoning in the receiving bodies [196]. Fe_3_O_4_/Au core–satellite nanocubes have been recently used to reduce PNP into para-aminophenol (PAP) [191], a product that has many useful applications such as analgesic drugs, photographic developer or even hair dyeing agents [197]. Other applications have made use of the AuNP dehydrogenase activity to degrade estradiol [190], which pollutes wastewaters and has been reported to feminize wild fish [198] and interfere with the human endocrine system [199].

### Food Safety Applications

In a similar manner to environmental applications, food diagnostics remains of paramount importance due to the implications that a contamination in this matrix has for human health. The array of contaminants that can be found in food is diverse, as much as the variety in food products. This includes micro-organisms, pesticides, antibiotics or toxins among others. The biomedical consequences of consuming contaminated food go from diarrhoea to cancer, potentially causing more than 200 diseases. According to the WHO (2019) an estimated 600 million people fall ill after consuming contaminated food, while 420,000 die every year as a result of it, being children under 5 years of age who mostly suffer these consequences [200]. Moreover, since food supply chains cross-national borders, any contamination can reach far more people than ever. Taking all this into account, diagnosing food contamination on time remains an issue of high biomedical relevance. Some gold nanozyme-based sensors have been developed targeting microbiological contaminants: *Escherichia coli* O157:H7 [201], *Pseudomonas aeruginosa* [187] and murine norovirus [186]. The first system is based on a sandwich-type immunochromatographic assay that uses Au–Pt bimetallic NPs to enhance the colour signal generated through their peroxidase-like activity. The second approach is based on the inhibition of the peroxidase-like activity of AuNPs: the NP are coated with a *Pseudomonas*-binding aptamer, which blocks the NP surface from developing the catalytic reaction, unless the target bacteria are present in the sample and sequester the aptamers, thus resuming the peroxidase activity. With the help of a disposable carbon screen-printed electrode, this simple system allows for the detection of 60 CFU mL^−1^ in 10 min. The same approach has been used to detect murine norovirus in shellfish homogenates, reaching an LOD of 200 PFU mL^−1^. However, the detection in this case is not electrochemical but colourimetric, showing the potential of this approach for point-of-care diagnostics, which remains an important goal considering that norovirus is the leading cause of viral foodborne outbreaks globally. In any case, this technology had been developed and applied for the detection of the pesticide acetamiprid [184] and the antibiotic kanamycin [202, 203] long before.

As mentioned in the previous section, children are especially sensitive to some of these contaminations. A clear example of this can be found in the melamine scandal happened in 2008 in China, when milk was illegally adulterated with this chemical. Melamine is a nitrogen-rich organic compound that has sometimes been illegally added to food products to increase the apparent protein content, thus masking a dilution in protein in the final product. This fraud should have been detected by routine tests implemented to ensure milk quality. However, these tests were either not carried out properly or ineffective [204, 205]. As a result, about 300,000 people were poisoned due to the high concentration of melamine, which caused many infants to develop kidney stones leading to renal failure and death of 6 babies [206]. Ever since, the number of publications related to melamine detection experienced a tenfold increase. Deng et al. [207] further demonstrated the versatility of gold nanozymes by developing a colourimetric sensor able to detect 0.02 mg L^−1^ of melamine in infant formula. This visual and straightforward system made use of the intrinsic binding of melamine to AuNPs to cause their aggregation and inhibit their peroxidase-like activity. This mechanism has the advantage of its simplicity, but as any sensor lacking a biorecognition element, the resulting specificity may be a matter of concern in complex matrixes. This LOD was reduced down to 8.51 nM by Li et al. [208], who used SERS properties of popcorn-like Au–Ag NPs to detect in a more sensitive manner the oxidized product generated by the bimetallic nanomaterial, oxTMB. They took advantage of the interaction between melamine and H_2_O_2_, which reduces the availability of the substrate for the peroxidase reaction of TMB, thus decreasing the colour generation in the presence of melamine. Interestingly, oxTMB is a Raman active compound, while anisotropic NPs are strong Raman enhancers [209, 210]. This can be exploited to detect the presence of melamine in milk powder with better sensitivity than the traditional colourimetric approach.

Similar frauds have occurred with different additives, not necessarily linked to fatal consequences. This is the case of unauthorized use of ascorbic acid (also known as vitamin C). In meat products, the ascorbic acid prevents the formation of nitrosamines that lead to the discolouration of the meat during storage [211], a sign of spoilage. In agreement with the “no adulteration” policy in this matter, Xu et al. [212] developed a sensor based on Au/Cu bimetallic NR for the determination of ascorbic acid in vitamin drink samples, reaching a LOD of 25 µM. Again, the inhibition of the peroxidase-like activity of AuNPs is the approach used, combined with the synergistic effect observed between Au and Cu, which results in a catalytic activity 4.5 times higher than Au NR.

**Fig. 12:**
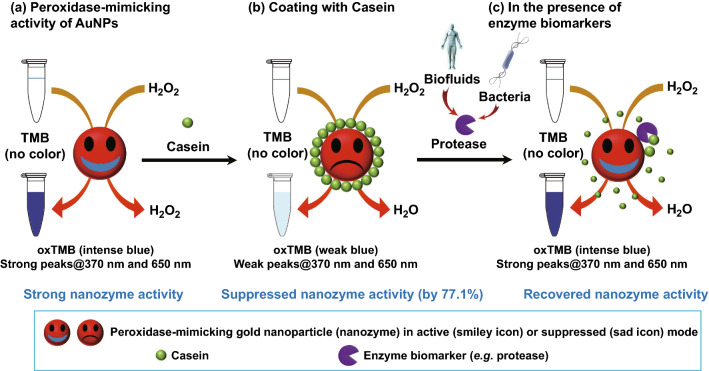
Colourimetric sensor using gold nanozymes for the detection of proteolytic biomarkers indicative of food spoilage or human disease [102]. Copyright 2018 McVey et al.

More sophisticate systems have taken advantage of this peroxidase-like activity of gold nanozymes using an indirect approach to detect the presence of a significant biomarker of a certain condition. McVey et al. [102] developed a gold nanozyme sensor to determine the presence of proteolytic enzymes in human urine, where they may be related to diabetic kidney disease if elevated, and in cow milk, where their presence may be indicative of an infection by micro-organisms causing bovine mastitis. Protein coating of the AuNP surface blocks the TMB oxidation, while the presence of protease in the sample hydrolyses the protein and resumes the peroxidase-like activity of the AuNPs (Fig. 12). Some of these environment- and food-related examples are summarized in Table 2.

**Table 2:** Other non-clinical biosensors relate the use of the catalytic properties of gold nanomaterials

Target	Nanomaterial	Activity	Outputs	Sample	LOD	References
Hg^2+^	g-C_3_N_4_–Au nanocomposites	Peroxidase	Colourimetric	Water	3 nM	[176]
Glucose	AuNPs	Glucose oxidase	Photoelectrochemical	–	0.46 µM	[229]
Hg^2+^	Au/Fe_3_O_4_/GO hybrid material	Peroxidase	Colourimetric	Water	0.15 nM	[177]
Acetamiprid	AuNPs	Peroxidase	Colourimetric	–	1.8 ppm	[184]
Kanamycin	AuNPs	Peroxidase	Colourimetric	–	1.49 nM	[202]
Glucose	Au–Ni/g-C_3_N_4_ nanocomposites	Peroxidase	Colourimetric	–	1.7 µM	[230]
Hg^2+^ and Ag^+^	Au–Pt core/shell NPs	Peroxidase (inhibition)	Colourimetric	Lake water	3.5 and 2 nM	[178]
Malathion	Pd–Au nanorods	Peroxidase (inhibition)	Colourimetric	Tap water	60 ng mL^−1^	[185]
Kanamycin	AuNPs	Peroxidase	Electrochemical	Standard and honey	0.06 and 0.73 nM	[203]
Nitrite	AuNCs/RGO nanocomposites	Peroxidase	Colourimetric and electrochemical	Sausages	2 and 0.7 µM	[231]
H_2_O_2_ and Glucose	AuNPs@DNA hydrogel	Peroxidase, glucose oxidase	Spectroscopic, colourimetric	Serum	1.7 and 38 µM	[232]
Malathion	Au nanorods	Peroxidase	Spectroscopic, colourimetric	Tap water	1.78 µg mL^−1^	[100]
Hg^2+^	AuNPs	Peroxidase	Colourimetric (smartphone)	Tap water	1.2 µg L^−1^ (≈ 6 nM)	[136]
H_2_O_2_	GO/AuNPs	Peroxidase	Spectroscopic and electrochemical	–	2 µM and 1.9 nM	[233]
Hg^2+^ and Pb^2+^	AuNPs/BiOI nanocomposites	Peroxidase	Fluorescence	Water (tap, river, lake, sea)	Nanomolar range	[179]
Ce^3+^	AuNPs	Peroxidase	Spectroscopic	Tap water	2.2 nM	[194]
Ag^+^	AuNPs	Peroxidase (inhibition)	Spectroscopic	Lake water	10 nM	[195]
Ascorbic acid	Au/Cu nanorods	Peroxidase	Spectroscopic	Vitamin drink	25 µM	[212]
Protease	AuNPs	Peroxidase	Colourimetric, spectroscopic	Milk	44 ng mL^−1^	[102]
Hg^2+^	AuNPs	Peroxidase	Colourimetric, spectroscopic	Water	0.3 nM	[180]
Melamine	Popcorn-like Au–Ag NPs	Peroxidase	Colourimetric, SERS	Milk powder	8.51 nM	[208]
Murine norovirus	AuNPs	Peroxidase	Colourimetric	Human serum, shellfish homogenate	200 PFU mL^−1^	[186]
*Pseudomonas aeruginosa*	AuNPs	Peroxidase	Electrochemical	Drinking water	60 CFU mL^−1^	[187]
*Escherichia coli* O157:H7	Pt–Au porous NPs	Peroxidase	Colourimetric	–	100 CFU mL^−1^	[201]

### Other Applications

**Fig. 13:**
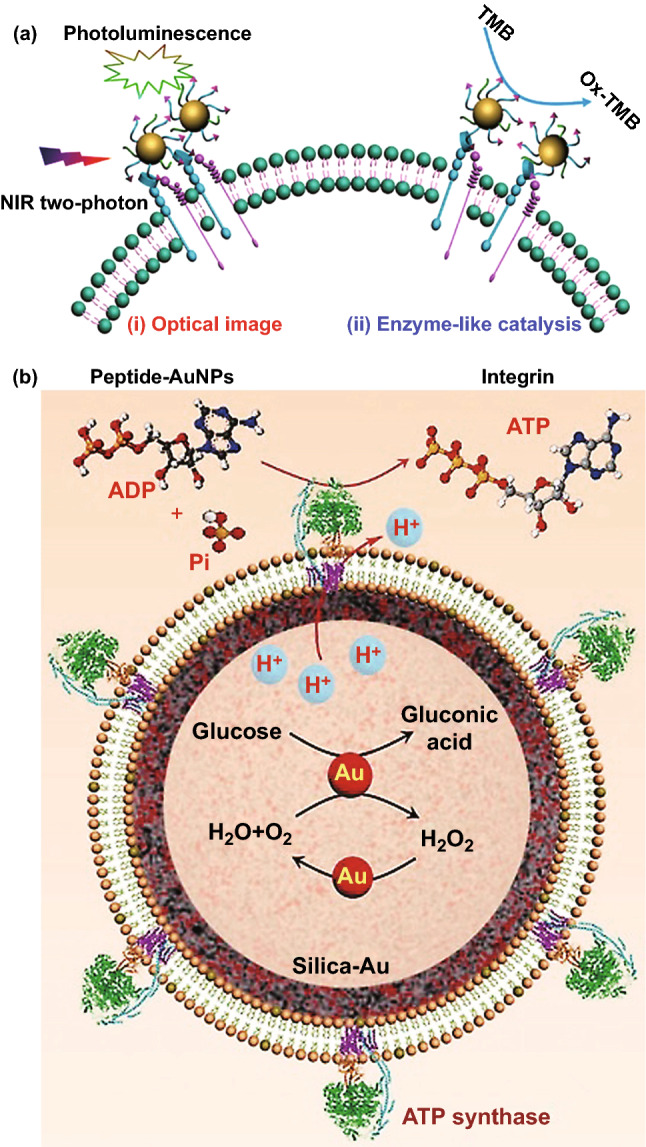
Cell membrane engineering applications are used for **a** diagnostics of a health condition [214]. Copyright 2015 American Chemical Society or for **b** mimicking the biological machinery of ATP production in an artificial complex [213]. Copyright 2019 Wiley–VCH Verlag GmbH & Co. KgaA, using in both cases the catalytic properties of AuNPs

Lastly, gold nanozymes have proved useful beyond diagnosis and treatment possibilities. Many studies have already shown some potential bioapplications in diverse fields worth mentioning, either for building artificial biochemical models [213, 214] or for implementing chemical catalysis [215, 216]. Regarding biochemical models, two of them have been developed using gold nanozymes with different applicabilities. The first one uses a ligand exchange process to prepare peptide conjugated AuNPs that can recognize integrin GPIIb/IIIa in the membrane of human erythroleukaemia cells [214]. This ex vivo system enables the detection and quantification of membrane protein expression levels that constitute an early diagnosis approach for cancer. Specifically, the mentioned integrin expression level is closely related to platelet aggregation and cancer pathogenesis. As shown in Fig. 13a, AuNPs can be used for direct optical imaging through photoluminescence (thus locating the target protein in the membrane) or for quantification by making use of their peroxidase-like activity to calculate the precise expression level, that may be linked to a disease condition. The second example of biochemical models consists in an assembled natural–artificial hybrid architecture containing gold nanozymes to simulate the mitochondria oxidative phosphorylation process [213]. This system, illustrated in Fig. 13b, uses the glucose oxidase-like activity of AuNPs immobilized on hollow silica microspheres to generate gluconic acid, thus lowering the pH value within an artificial proteoliposome membrane containing ATP synthase. The proton gradient generated is then used to drive the rotary catalysis of ATP synthase, converting ADP and inorganic phosphate into ATP. However, there is a key drawback in this system, which is the generation of H_2_O_2_ as a secondary product. This could lead to the oxidation of the enzyme through ROS generation, whereas the potential effects of this H_2_O_2_ generation could have far more implications in vivo [217]. The duality of gold nanozymes helps to solve this problem through their peroxidase-mimicking activity, which in the presence of H_2_O_2_ and HAuCl_4_ results in the continuous enlargement of the AuNPs.

**Fig. 14:**
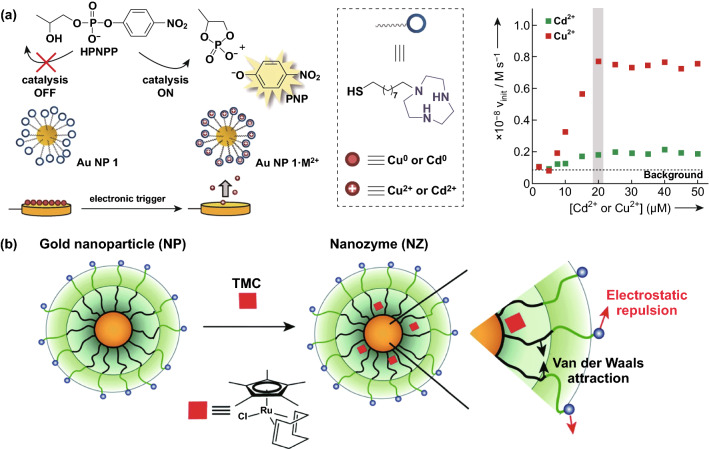
Traditional nanozyme definition combining AuNPs with metallic ions used as catalysts. **a** Catalytic cleavage of HPNPP by Cd^2+^ and Cu^2+^ ions complexed by the AuNP-bound monolayer [216]. Copyright 2016 Wiley–VCH Verlag GmbH & Co. KGaA. **b** Ru catalysts inserted in the protective layer of AuNPs [215]. Copyright 2017 The Royal Society of Chemistry

Finally, several supramolecular nanosystems that take part in more complex chemical reactions have also been reported. However, many of these examples use AuNPs as scaffolds in which the catalysts are immobilized. Metallic ions are commonly used for this purpose, such as Cd^2+^ and Cu^2+^ [216] or Ru [215]. These models follow the traditional nanozyme definition, coined by Scrimin’s group [76], and referring to the combination of nanomaterials and catalysts, with no evaluation of the functionality of the nanomaterial itself as catalyst. The first nanosystem catalyses the cleavage of 2-hydroxypropyl-p-nitrophenyl phosphate (HPNPP) [216] and demonstrates that the activity of this supramolecular catalyst can be electrochemically regulated by releasing metallic cations from an electrode or by re-depositing these ions on the electrode (Fig. 14a). The second example uses biorthogonal ruthenium catalysts inserted in the protective monolayer of AuNPs [215] (Fig. 14b) to activate the low-fluorescent allyl carbamate-protected rhodamine into its fluorescent counterpart.

These are some examples showing important and useful applications of gold nanozymes. However, some other possibilities remain relatively undiscussed for being in a very primitive stage or lacking the desired portability, as many SERS studies on gold nanozymes show [218–221]. Thus, the applications in this field are expected to quickly grow in the near future, being the ones herein some exemplifications of the most pioneer approaches.

## Conclusions and Future Perspectives

The diversity of applications described so far (biosensors for biomedical, environmental or food applications, cancer treatment, cellular protection against conditions causing increased ROS levels, catalysis of many reactions used in the chemical industry, etc.) show the versatility and potential of gold nanozymes. Moreover, only the applications with some medical, biological and environmental relevant aspects have been discussed in this review. However, several gaps have been identified in the area of gold nanozymes that need to be solved before a further development of the area can be achieved.

The first observation that could diminish the potential of gold nanozymes if not considered would be the lack of robustness of these systems when their surface is chemically modified in any way. There is clear evidence that the immobilization of stabilizing agents on the surface of the AuNPs entails a modification of the catalytic properties of the nanosystem in question, most of the times resulting in the inhibition of the process due to the blockage of the gold surface where the reaction happens. This problem aggravates when the coating requires macromolecules (such as antibodies or other biological binders), leading to a complete inhibition of the catalytic process. Because of this, less than one quarter of the studies found in the searching databases using the term “gold nanozyme” make use of any sort of biorecognition macromolecule attached to the AuNPs, whereas all the others prefer to follow an approach that does not require much of a modification in the NP surface. The latter scenario, where no biorecognition elements are used, follows alternative approaches where the stabilizing agent of the nanomaterials (often polymers, detergents or other low molecular weight agents) shows some specificity towards a certain target analyte. Despite this approach being effective in a number of cases reported throughout the review, there are valid concerns about their specificity in real complex matrixes. Among the approaches that use immobilized biorecognition elements, most studies chose either aptamers or antibodies. In the first case, an approach that has gained popularity and that has been adapted in many systems consists in coating the AuNPs with an aptamer to inhibit the peroxidase-like activity of AuNPs and then restore it if the aptamer is released and captured by the presence of its target analyte. In the case of antibodies, an interesting approach to solve the inhibition consists in implementing a metallic deposition on the nanoconjugate (i.e., gold or silver deposition) to restore the catalytic properties lost during the surface modification [222]. Nevertheless, there are still many studies that have proved the potential of certain applications using bare AuNPs and never tested their applicability in real conditions where the surface modification is mandatory. This shows the first obstacle that must be overcome by gold nanozymes. Logically, the above explanation leads to another question: can the gold nanozyme-based systems be affected by the diversity of molecules present in the real matrixes? This is an interesting and valid question whose answer is difficult to predict and will depend on case to case. This might limit the potential of these systems for in vivo applications, where the nanosystems have to be administrated into the body while retaining their catalytic properties. Ex vivo applications, on the other hand, might not experience this problematic as the possibility of sample pre-treatment could avoid it.

Considering in vivo applications, which shape a big block within biomedical applications of gold nanozymes, the second obstacle that arises is the lack of information about toxicity and biodistribution of AuNPs. Nanomaterials used as pharmaceutical drugs must also be eliminated via metabolism or excretion processes after they enter the body, to reduce toxicity and prevent drug accumulation. Even if AuNPs are not inherently toxic to human cells, it has been evident that some precursors and capping molecules might cause toxicity. Previous studies using quantum dots in mice showed that these nanomaterials remained intact for more than 2 years in mouse tissues [223], which is a health and safety concern. Together with the lack of information about long-term effects of the presence of NP in human tissues, their applicability for health issues is stagnant. Nanomaterials whose hydrodynamic diameter is smaller than 5 nm will be excreted by the renal route, whereas bigger nanomaterials will not traverse the glomerular filter and thus will accumulate in spleen and liver [118, 200, 224]. The complexity of these studies leads to some biomedical applications of gold nanozymes being studied in vitro, such as the neuronal protection against oxidative stress illustrated in Fig. 10a, whose applicability in vivo still seems distant.

Furthermore, as it has been previously mentioned, AuNPs are extraordinarily versatile enzyme mimics, showing peroxidase activity, glucose oxidase, catalase, SOD or even reductase. More importantly, these activities coexist in some cases. However, there is a lack of information on what conditions favour or inhibit each of them, existing only very basic information related to the pH of the media to help explain this phenomenon. Finally, there is only one publication reporting to have combined gold nanozymes and smartphones in any way. Both gold nanozymes as transduction approach and smartphones as quantification devices are novel tools, which highlights the novelty of this area and the potential that it could have for portable diagnostics once it starts to develop.

**Fig. 15:**
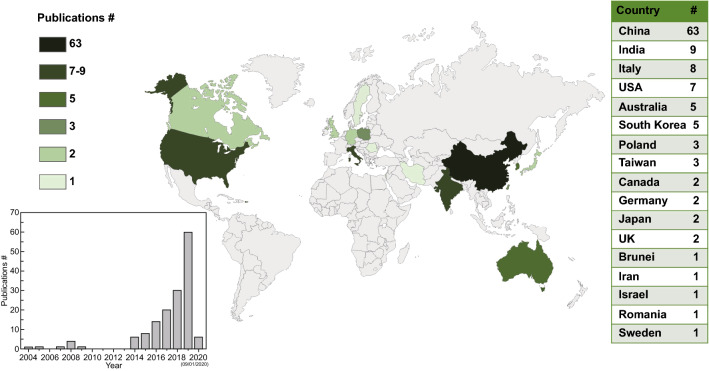
Worldwide distribution of gold nanozyme publications, top-producing countries in the field and annual progression in the field by number of publications

All the points described in this section, in which the obstacles that one may encounter when developing a nanozyme-based device are summarized, intend to convey useful and critical information extracted from the current state of the research rather than to discourage future works. As it is illustrated in the map (Fig. 15), only 17 countries have published research articles on gold nanozymes, which means the area is yet entirely to explore. More than half of these research activities were conducted in China, and only 8 articles containing the term “gold nanozyme” were published before 2014. All this is indicative of the novelty of the area, which according to Fig. 15 seems to be in an exponential growth phase. Therefore, the potential commercialization of the assays and therapies discussed herein would need more time to be realized and to be evaluated in order to bring the cutting-edge technologies to commercial market.

In summary, over 200 recently published research papers were systematically reviewed to present the current progress on the fundamentals of gold nanozymes and their potential applications. The review reveals that the morphology and surface chemistry of the nanoparticles play an important role in their catalytic properties, as well as external parameters such as pH or temperature. Yet, real applications often require specific biorecognition elements to be immobilized onto the nanozymes, leading to unexpected positive or negative effects on their activity. Thus, rational design of efficient nanozymes remains a challenge of paramount importance. Different implementation paths have already been explored, including the application of peroxidase-like nanozymes for the development of clinical diagnostics or the regulation of oxidative stress within cells via their catalase and SOD activities. The review also indicates that it is essential to understand how external parameters may boost or inhibit each of these activities, as more than one of them could coexist. Likewise, further toxicity studies are required to ensure the applicability of gold nanozymes in vivo. By providing insights into the fundamental aspects, applicability and difficulties of the gold nanozyme-based systems, this review is to provide an in-depth yet informative investigation. Although several concepts have been widely exploited, more studies are required to validate whether the approaches observed can be applied in real situations.
